# Exercise training improves aerobic endurance and musculoskeletal fitness in female cardiac transplant recipients

**DOI:** 10.1186/1468-6708-6-10

**Published:** 2005-05-26

**Authors:** Mark Haykowsky, Kenneth Riess, Linda Figgures, Daniel Kim, Darren Warburton, Lee Jones, Wayne Tymchak

**Affiliations:** 1 Faculty of Rehabilitation Medicine, University of Alberta, Edmonton, Alberta, Canada; 2 Division of Cardiology, Faculty of Medicine, University of Alberta, Edmonton, Alberta, Canada; 3 School of Human Kinetics, University of British Columbia, Vancouver, British Columbia, Canada; 4 Program of Cancer Prevention Detection and Control, Duke University Medical Center, Durham, North Carolina, USA

## Abstract

**Aim:**

Female cardiac transplant recipients' aerobic capacity is 60% lower than sex and age-predicted values. The effect of exercise training on restoring the impaired aerobic endurance and muscle strength in female cardiac transplant recipients is not known. This study examined the effect that aerobic and strength training have on improving aerobic endurance and muscle strength in female cardiac transplant recipients.

**Methods:**

20 female cardiac transplant recipients (51 ± 11 years) participated in this investigation. The subjects performed a baseline six-minute walk test and a leg-press strength test when they were discharged following cardiac transplantation. The subjects then participated in a 12-week exercise program consisting of aerobic and lower extremity strength training. Baseline assessments were repeated following completion of the exercise intervention.

**Results:**

At baseline, the cardiac transplant recipients' aerobic endurance was 50% lower than age-matched predicted values. The training program resulted in a significant increase in aerobic endurance (pre-training: 322 ± 104 m vs. post-training: 501 ± 99 m, p < 0.05) and leg-press strength (pre-training: 48 ± 16 kg. vs. post-training: 78 ± 27 kg, p < 0.05).

**Conclusion:**

Aerobic and strength training are effective interventions that can partially restore the impaired aerobic endurance and strength found in female cardiac transplant recipients.

## 

Exercise training is an effective intervention that can partially restore the impaired aerobic capacity and musculoskeletal fitness (i.e. muscle strength) found in cardiac transplant recipients [1,2]. However, previous reports have focused exclusively on the effects of exercise training in men. Therefore, the effect of exercise training on these outcomes in female cardiac transplant recipients is not known [2-7]. Importantly, a majority of female cardiac transplant recipients do not engage in regular physical activity leading to increased levels of fatigue, poor functional status and reduced exercise capacity [8-10]. Based on this rationale, the aim of this study is to examine the effect that exercise training has on improving aerobic endurance (i.e. distance walked in six-minutes) and lower extremity muscle strength in female cardiac transplant recipients. We hypothesized that exercise training would be a feasible and effective intervention to improve aerobic endurance and lower extremity strength in female cardiac transplant recipients.

## Methods

### Subjects and procedures

The participants for this study consisted of 20 (51 ± 11 years) clinically stable female cardiac transplant recipients who participated in the University of Alberta Post-Transplant Exercise Rehabilitation program between 1997 and 2003. All assessments and exercise training were performed in the Physical Therapy Department at the University of Alberta Hospital. Ethics approval for this study was obtained from the Biomedical Ethics Board at our University.

### Outcome Assessments

The six-minute walk test was performed in accordance with the American Thoracic Society guidelines [11]. In addition, the six-minute walk scores were compared with age-matched norms for healthy females published by Gibbons and associates [12]. Leg-press maximal strength testing was performed on a commercially available leg-press machine with the greatest weight lifted while adhering to strict technique being used as the maximal score. All assessments were repeated following the 12-week training program.

### Exercise Training Intervention

Exercise training consisted of supervised aerobic (cycling and/or treadmill exercise at an intensity between 12 to 14 on the BORG perceived exertion scale for 30 to 40 minutes/day including warm-up and cool-down, 5 days/week) and lower extremity strength training.

### Data analysis

Statistical analysis was performed with a one-way analysis of variance. The alpha level was set "a priori" at p < 0.05. Data are presented as mean ± SD.

## Results

Baseline testing was performed 37 ± 27 days after cardiac transplantation.

### Aerobic Endurance

**Figure 1:**
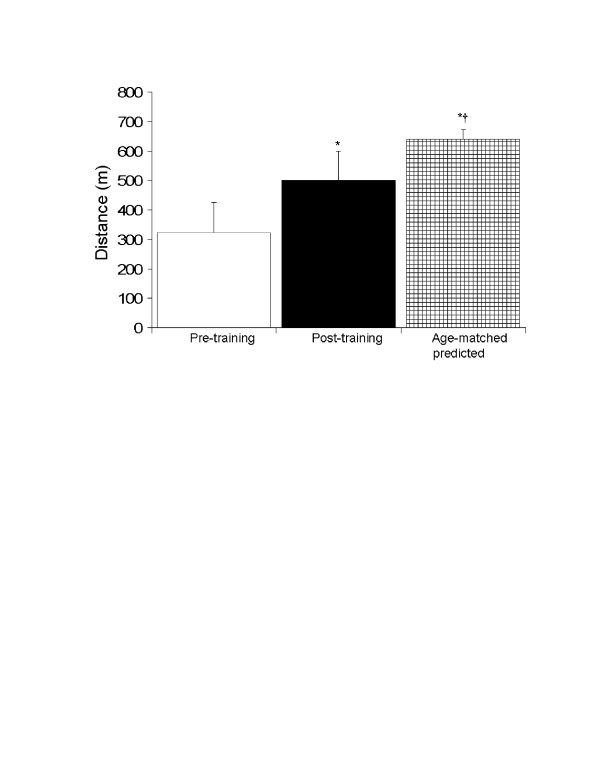
Effect of exercise training on the distance walked in six-minutes. *, p < 0.05 vs. pre-training; ^†^, p < 0.05 vs. post-training.

At baseline, our participants' aerobic endurance was 50% lower than age-matched predicted values (Figure 1). Twelve weeks of training resulted in a significant increase in aerobic endurance, however, it remained 22% lower than age-predicted values (Figure 1).

### Leg Press

Leg-press maximal strength increased by 64% after three months of training (pre: 48 ± 16 kg vs. post: 78 ± 27 kg, p < 0.05).

## Discussion

This is the first study to examine the effect that combined aerobic and strength training have on improving aerobic endurance and musculoskeletal fitness in female cardiac transplant recipients. The main finding of this study is that combined aerobic and strength training is a feasible and effective intervention to partially restore female cardiac transplant recipients' aerobic endurance and leg-press strength.

Cole et al. [9] recently found that female cardiac transplant recipients' aerobic capacity was 60% lower than age-predicted values. Consistent with this finding, our transplant recipients' baseline aerobic endurance was 50% lower than age predicted values. Moreover, our participants pre-training leg-press strength was 36% lower than that found in age-matched male cardiac transplant recipients tested in our laboratory [13]. The mechanisms responsible for the impaired cardiovascular and musculoskeletal fitness are likely secondary to abnormalities in cardiac and skeletal muscle function associated with pre-transplant heart failure, post-transplant deconditioning, cardiac denervation or immunosuppressuion therapy [14].

Several research groups [2,3,13] have demonstrated that exercise training initiated in the early post-operative period is associated with an increase aerobic endurance [13], muscle mass [2], muscle strength [2,13] and bone density [3] in male cardiac transplant recipients. This study extends previous investigations by demonstrating that 12 weeks of combined aerobic and strength training are associated with a significant and marked improvement in aerobic endurance and muscle strength in female cardiac transplant recipients. The mechanisms responsible for the improvement in aerobic endurance was not examined in this study, however, they may be due to favorable improvements in mitochondrial oxidative properties [15,16] that increase arteriovenous oxygen difference during exertion as aerobic training does not alter exercise cardiac output in this population [4,17]. The training mediated increase in leg-press strength that we found is likely secondary to the increase in muscle mass that occurs with aerobic [4] or combined aerobic and strength training [2]. The consequence of our training mediated improvement in cardiorespiratory and musculoskeletal fitness is that it may result in a favorable improvement in mortality. Specifically, Kavanagh et al. [18] reported that cardiac transplant recipients with the greatest training-mediated improvement in aerobic capacity and lean body mass had a lower mortality rate 12 years after cessation of the training program.

A limitation of our investigation is that we did not have a non-exercise control group. However, our cardiac transplant recipients are required to participate in a supervised 12-week exercise program beginning as an inpatient and completed as an outpatient. Despite this limitation, the improvement in aerobic endurance and leg-press strength associated with our training program is similar to that found in male cardiac transplant recipients who participated in our outpatient exercise rehabilitation program [13].

## Summary

A majority of female cardiac transplant recipients adhere to a sedentary lifestyle and as result their aerobic capacity is 60% lower than age-predicted values [9]. The effect that exercise training has on improving female cardiac transplant recipients' aerobic endurance and lower extremity strength is not known. The primary finding of this investigation is that 12 weeks of aerobic and strength training is an effective intervention that can improve aerobic endurance and musculoskeletal fitness in recent female cardiac transplant recipients. Moreover, the improvement in aerobic endurance and leg-press strength is similar to that found in male cardiac transplant recipients after combined aerobic and strength training. In summary, female cardiac transplant recipients should be encouraged to perform aerobic and strength training to increase their aerobic endurance and musculoskeletal fitness.

## Competing interests

The author(s) declare that they have no competing interests.

## Authors' contributions

MH. Conceived the study, performed data analysis and manuscript preparation.

KR. Assisted with data collection and manuscript preparation.

LF. Performed the exercise rehabilitation training and assisted with manuscript preparation.

DK. Assisted with manuscript preparation.

DW. Assisted with manuscript preparation.

LJ. Assisted with manuscript preparation.

WT. Assisted with manuscript preparation.

All authors read and approved the final manuscript.
